# AIM2 inflammasome activation benefits the therapeutic effect of BCG in bladder carcinoma

**DOI:** 10.3389/fphar.2022.1050774

**Published:** 2022-10-31

**Authors:** Houhong Zhou, Lei Zhang, Weihan Luo, Huaishan Hong, Dongdong Tang, Dewang Zhou, Lingli Zhou, Yuqing Li

**Affiliations:** ^1^ Institute of Urology, The Third Affiliated Hospital of Shenzhen University, Luohu Hospital Group, Shenzhen University, Shenzhen, China; ^2^ Luohu Clinical Medicine School, Shantou University Medical College, Shantou University, Shantou, China; ^3^ Department of Urology, Fujian Provincial Hospital, Fuzhou, Fujian, China; ^4^ South China Hospital, Health Science Center, Shenzhen University, Shenzhen, China

**Keywords:** bladder carcinoma, AIM2, inflammasome, BCG immunotherapy, progression

## Abstract

A large proportion of bladder cancer (BLCA) patients suffer from malignant progression to life-threatening muscle-invasive bladder cancer (MIBC). Inflammation is a critical event in cancer development, but little is known about the role of inflammation in BLCA. In this study, the expression of the innate immune sensor AIM2 is much lower in high-grade BLCA and positively correlates with the survival rates of the BLCA patients. A novel AIM2 overexpressed BLCA model is proposed to investigate the impact of AIM2 on BLCA development. Mice inoculated with AIM2-overexpressed cells show tumor growth delay and prolonged survival compared to the control group. Meanwhile, CD11b^+^ cells significantly infiltrate AIM2-overexpressed tumors, and AIM2-overexpression in 5637 cells enhanced the inflammasome activation. In addition, oligodeoxynucleotide (ODN) TTAGGG (A151), an AIM2 inflammasome inhibitor, could abolish the elevation of AIM2-induced cleavage of inflammatory cytokines and pyroptosis. Orthotopic BLCA by AIM2-overexpressed cells exhibits a better response to Bacillus Calmette-Guérin (BCG) immunotherapy. Overall, AIM2 inflammasome activation can inhibit the BLCA tumorigenesis and enhance the therapeutic effect of BCG in BLCA. This study provides new insights into the anti-tumor effect of AIM2 inflammasome activation in BLCA and the immunotherapeutic strategy of BLCA development.

## Introduction

Bladder cancer (BLCA) is the most common malignancy of the urinary system and is associated with high morbidity and mortality despite chemotherapy (Tran et al., 2021). BLCA can be classified as non-muscle-invasive bladder cancer (NMIBC) and muscle-invasive bladder cancer (MIBC), depending on the depth of invasion (Tran et al., 2021). NMIBC accounts for approximately 80% of BLCA cases (Sanli et al., 2017; Bray et al., 2018). Traditional surgical resection followed by immunotherapy with intravesical Bacillus Calmette-Guérin (BCG) vaccine or intravesical chemotherapy has been the mainstay of treatment for NMIBC (Han et al., 2020). Although NMIBC possesses limited metastatic and/or lethal potential, it shows a high recurrence rate, and approximately 25%–30% of NMIBC may progress to MIBC. Typically, MIBC progresses rapidly to metastatic disease and has a high mortality rate, and patients with MIBC would receive treatment involving radical cystectomy or transurethral resection with chemotherapy (Sanli et al., 2017; Bray et al., 2018). Therefore, patients with BLCA have to experience the burden of lifelong cystoscopic surveillance and multiple therapeutic interventions, leading to significant financial and emotional stress (Sanli et al., 2017; Sloan et al., 2020). Immune checkpoint inhibitor therapeutics provide long-lasting clinical benefits to patients with BLCA. However, only a fraction of patients respond to this treatment (Tran et al., 2021).

Inflammation plays a complex role from cancer initiation to its progression and deterioration (Grivennikov et al., 2010). However, the molecular pathways involved in inflammation-related BLCA remain largely uncertain (Shadpour et al., 2019). Absent in melanoma 2 (AIM2), a receptor for cytosolic double-stranded DNA (dsDNA) of pathogens-associated or host origin is composed of an N-terminal pyrin domain and a C-terminal HIN-200 domain (Broz and Dixit, 2016; Kumari et al., 2020). The two domains usually form an intramolecular complex to make AIM2 maintaining in an auto-inhibitory state during homeostasis (Broz and Dixit, 2016). Once AIM2 senses cytoplasmic DNA, it recruits the adaptor protein apoptosis-associated speck-like protein containing a CARD (ASC). ASC recruits pro-caspase-1 to assemble a macromolecular complex termed the inflammasome, which induces caspase-1 activation (Kumari et al., 2020). Afterward activated caspase-1 leads to cleavage and release of the inflammatory cytokines interleukin 1β (IL-1β) and interleukin 18 (IL-18), and pore-forming protein gasdermin-D (GSDMD)-mediated pyroptosis, which eventually triggers the initiation of an immune response (Kumari et al., 2020; Wang et al., 2020). Although it is generally acknowledged that inflammation plays a critical role in cancers, the effect of the AIM2 inflammasome in tumorigenesis is still unclear (Coussens and Werb, 2002; Grivennikov et al., 2010; Greten and Grivennikov, 2019). Initially, AIM2 was suspected to be a tumor suppressor gene in melanomas (DeYoung et al., 1997). Kong et al. reported that the overexpression of AIM2 could promote AIM2 inflammasome formation and activation in hepatocarcinoma cells (Kong et al., 2015). In contrast, AIM2 functioned as an oncogene in Non-small-cell lung cancer (NSCLC) in an inflammasome-dependent way (Zhang et al., 2019). AIM2 is also reported to be a tumor suppressor or oncogene in inflammasome-independent manner (Wilson et al., 2015). Therefore, the mechanism of AIM2 affects BLCA should be investigated based on the speculation of AIM2 may work in BLCA.

In this study, we indicated that AIM2 is lowly expressed in high-grade BLCA, and the expression of AIM2 is markedly correlated with the overall survival rates in BLCA patients. We further verified AIM2 inflammasome as a cancer suppressor in BLCA both *in vitro* and *in vivo*. Mice inoculated with AIM2-overexpressed MBT-2 cells show a prolonged survival rate, smaller tumor size, lighter tumor weight, and more infiltration by CD11b^+^ cells compared to the control. In addition, AIM2-overexpression in 5637 cells enhanced the agonist poly (dA:dT)-induced ASC polymerization, caspase-1 activation, IL-1β and IL-18 secretion, and pyroptosis. Moreover, BCG immunotherapy for orthotopic BLCA is more effective in AIM2-high-expressed tumors. These findings reveal a previously unappreciated role of AIM2 inflammation activation in BLCA cells in restricting cancer progression and suggest that AIM2 inflammasome activation could be applied to BLCA immunotherapeutic strategy.

## Materials and methods

### Clinical specimens

Human BLCA samples and paraffin-embedded tissue sections were obtained from the 3rd Affiliated Hospital of Shenzhen University, Shenzhen Luohu Hospital Group, in compliance with the informed consent policy. The use of the tissue samples was approved by the Institutional Review Boards of Luohu Hospital Group.

### Cell lines and culture conditions

The murine bladder cancer cell line MB49 was purchased from Emd Millipore (Merck, cat# SCC148) and MBT-2 was obtained from the First Affiliated Hospital of Anhui Medical University. The human BLCA cell line 5637 and MGH-U3 were purchased from the American Type Culture Collection (ATCC) and Leibniz-Institut Deutsche Sammlung von Mikroorganismen und Zellkulturen GmbH (DSMZ), respectively. MB49 cells were cultured in DMEM basic (8122305, Gibco; Waltham, MD, United States) containing 10% (v/v) Fetal bovine serum (FBS). Other cell lines were cultured in RPMI-1640 (22400089, Gibco; Waltham) containing 10% (v/v) FBS. Both cell lines were incubated with 5% CO_2_ at 37°C.

### Cell lines constructs

For stable knock-in, the full-length mAIM2 or AIM2 was cloned into a lentivector-based FG-EH-DEST for lentivirus production using the Gateway Cloning System (Invitrogen). Plasmids with packaging plasmids psPAX2 and pMD2. G were transfected into HEK293T cells using polyetherimide for lentivirus production. Lentivirus particles were harvested at 48 h and 72 h after transfection and concentrated by ultracentrifugation. Finally, concentrated lentivirus particles were added to MB49, MBT-2, or 5637 cells for 6 h and replaced with fresh culture medium. To isolate mAIM2, AIM2 or luciferase knock-in clones, transduced cells were selected by treatment with 0.5–5 μg/ml puromycin for 3–4 days, then diluted to one cell per 100 µl media and plated into the wells of a 96-well plate. Single cell-derived colonies were expanded and subjected to immunoblotting (IB) validation.

### Cell stimulation, cytokine analysis and LDH cytotoxicity assay

For experiments determining AIM2 activation, cells were treated with or without ODN TTAGGG (A151) (InvivoGen, tlrl-ttag151) and incubated for an hour before stimulation. Then poly (dA:dT) at a concentration of 1 μg/ml was transfected using Lipofectamine^TM^ 3000 reagent (L3000008, Invitrogen) and Opti-MEM (31985088, Gibco) for 9 h. Cells were collected to determine precursor forms of caspase-1, Gasdermin D (GSDMD), and IL-1β. Cleavaged caspase-1, GSDMD, and IL-1β in culture supernatants were detected using IL-1β ELISA (557953, BD OptEIA^TM^), IL-18 ELISA (K2063302, Labtache), and LDH cytotoxicity assay kit (ab65393, abcam), according to the manufacturer’s instructions.

### Supernatant precipitation

5637 cells were seeded in a six-well plate to achieve protein precipitation from the supernatant. After the cells were attached to the six-well plate, the related cell culture medium was substituted by Opti-MEM medium (11058021, Gibco^TM^) before cells were treated with or without poly (dA:dT) stimulation. The supernatants were collected by centrifugation at 800 *g*/4°C for 5 min. Supernatants of each sample were precipitated with methanol/chloroform (supernatants/methanol/chloroform with a volume ratio of 4:4:1), then vortexed and centrifuged at 16,000 g/4°C for 15 min. Discard the upper supernatant and add the same volume of methanol. Vortexed and centrifuged at 16000 g/4°C for 15 min. Discard the supernatant, and the protein pellets were dried at 65°C for 5 min. Pellets were dissolved with 25 µl of 2× loading buffer, incubated at 100°C for 10 min, and resolved by IB.

### Immunoblotting

Tumor cells were lysed in RIPA buffer (25 mM Tris-HCl pH 7.6, 150 mM NaCl, 1% NP-40, 1% sodium deoxycholate, 0.1% SDS) containing phosphatase inhibitor cocktail (524629, Roche; Basel) and protease inhibitor cocktail (B14012, Bimake; Shanghai) on ice for 30 min, and then centrifuged at 12,000 g at 4°C for 10 min. The supernatants were collected, and the protein concentration was quantified by the BCA method. Protein lysates (20 μg per lane) were separated by SDS-PAGE and transferred onto nitrocellulose membranes. Membranes were blocked in 5% milk for 1 h, followed by overnight incubation with primary antibodies at 4°C. Membranes were washed for 3 × 10 min at room temperature in Tris-buffered saline–Tween 20 (TBST). Mouse or rabbit HRP-conjugated secondary antibodies were incubated for 1 h in 5% milk at room temperature, followed by washing 3 × 10 min at room temperature in TBST. Proteins were detected using Immobilon Western HRP Substrate (WBKLS0500, Millipore, Darmstadt).

The primary antibodies used in this study including: Caspase-1/p20 (1:1000, 2225; Cell Signaling Technology), IL-1β (1:1000, 12242; Cell Signaling Technology), Gasdermin D (1:1000, 39754; Cell Signaling Technology), ASC/TMS1 (1:1000, 10500-1-AP; Cell Signaling Technology), Flag (1:4000, 635 80010-1-RR, Proteintech), AIM2 (1:1000, DF3514, Affinity), Tublin (1:10000, 210324, Affinity) and GAPDH (1:10000, 60004-1-Ig, Proteintech).

### Immunofluorescence staining

Tumor cells were fixed in 100% methanol (chilled at −20°C) for 5 min and then washed three times with ice-cold PBS. Cells were then incubated in PBST (PBS +0.1% Tween 20) containing 1% BSA and 10% goat serum for 30 min to block the unspecific binding of the antibodies. Incubate cells with anti-ASC antibody (1:200, 10500-1-AP; Cell Signaling Technology) in PBST containing 1% BSA in a humidified chamber overnight at 4°C. Decant the first primary antibody solution and wash the cells 5 min for three times with PBS. Incubate cells with IFluor 488 (HA1121, HuaBio) in PBST containing 1% BSA for 1 h at room temperature in the dark. Decant the first secondary antibody solutions and wash the cells three times with PBS for 5 min each in the dark. Incubate cells with DAPI (AR1177, BOSTER) for 1 min and rinse with PBS. Images were photographed by confocal laser scanning microscopy (LSM800; Carl Zeiss) using ×63 Apochromat objective in a blinded manner and analyzed with ImageJ software (https://imagej.net/).

### BCG preparation

BCG (Chengdu Institute of Biological Products Co.,Ltd.) was grown at 37°C in Middlebrook 7H9 supplemented with 10% albumin/dextrose/saline, 0.5% glycerol, and 0.05% Tween 80. To create titered stocks for infection, BCG was grown to mid-log phase (OD_600_ 0.4–0.6), washed twice with PBS, resuspended in PBS with 25% glycerol, and stored at −80°C. An aliquot was thawed, and serial dilutions were cultured on 7H10 agar to measure the final bacterial titer. The bacterial titer was then determined by counting colonies after 3–4 weeks of incubation.

### Subcutaneous model, mouse orthotopic bladder cancer model, and intravesical BCG treatment

All animal experiments were conducted following protocols approved by the Luohu People’s Hospital’s Ethics committee and the ethical regulations regarding animal research. The subcutaneous model utilized NCG mice, and orthotopic transplanted and BCG studies used C57BL/6 mice. All mice were purchased from Guangdong Medical Laboratory Animal Center and raised under specific pathogen-free conditions.

Female mice between six and eight weeks of age were used for the subcutaneous model. Mice were transplanted with 1 × 10^6^ MBT-2 cells resuspended in 100 μl of a mixture (5:1) of serum-free RPMI-1640 with Matrigel (354248, Corning, NY) subcutaneously into the right flanks. Subcutaneous tumor growth was measured by calipers, and the volume was calculated using the formula: volume = (length × width^2^)/2.

For the orthotopic model, BLCA cell lines were trypsinized and resuspended in a mixture (5:1) of serum-free RPMI-1640 (Gibco) with Matrigel (BD Biosciences). Cells within a 25 μl suspension were transplanted into the bladder walls; details of the experimental procedure are available in (Fu et al., 2011). 6∼8 weeks female C57BL/6 mice were anesthetized with an intraperitoneal injection of pentobarbital sodium salt (Y0002194, Sigma-Aldrich). Mice were cleaned, and the surgical site was disinfected with the povidone-iodine solution. The bladder was exposed through a 1-cm midline incision, and urine was aspirated from the bladder. Subsequently, a 31- gauge needle with a syringe was utilized to inject 25 μl of cell suspension into the bladder wall. After the injection, remove the syringe and push the exposed bladder back into the abdomen with gentle pressure. Finally, the muscle layers and the skin were sutured with 6.0 resorbable sutures.

Frozen titered stocks of BCG were thawed and resuspended in PBS for a final concentration of 3 × 10^7^ colony forming units/ml (CFU/ml), and the control group used PBS alone. Mice were put under anesthesia with an intraperitoneal injection of pentobarbital for intravesical treatment. A 24-gauge catheter (Terumo) was inserted into the bladder through the urethra, and 50 μl of BCG (∼3 × 10^6^ CFU per mouse) or PBS was injected into the bladder.

After orthotopic transplantation, mice were injected with D-luciferin potassium salt (GM-040621, Genomeditech) at different point of time and imaged using an IVIS spectrum *in vivo* imaging system to evaluate tumor size in the bladder. The tumor uptake rates of all experiments are 100%.

### Immunohistochemistry staining

Tumors were resected and fixed in 4% paraformaldehyde overnight, and all paraffin-embedded materials were sectioned at 6 μm. After deparaffinization and rehydration, sections were subjected to antigen retrieval using 10 mM sodium citrate (pH 6.0) buffer for 10 min with an electric pressure cooker. Sections were then blocked with 3% hydrogen peroxide (H_2_O_2_) containing 0.1% Triton X-100 for 10 min to quench endogenous peroxidase activity. For regular immunohistochemistry, slides were stained overnight with primary antibodies in blocking buffer at 4°C, rinsed, and then incubated with goat anti-mouse/rabbit Horseradish Peroxidase (HRP)-conjugated secondary antibodies at 1:200 dilution in PBS for 30 min at room temperature. Staining was visualized with diaminobenzidine tetrahydrochloride (DAB) chromogen. Slides were washed in distilled water, counterstained with hematoxylin, dehydrated, and then mounted with permanent media. For sections stained with hematoxylin-eosin (H&E) and regular IHC, slides were screened with a digital scanner (KF-PRO-005, KFBio) and analyzed by K-viewer 1.5.3.1 software.

For multiplex immunohistochemistry (MIHC), primary antibodies were incubated sequentially according to the instructions of the Multiplex IHC kit (6-color, Panovue). As noted previously, deparaffinization, rehydration, antigen retrieval, and blocking were performed following robust laboratory workflows. After primary and secondary antibody incubation and washing 3 times with PBS, fluorescein for different absorption wavelengths (520, 570, and 650) was added to the sections in 1:100 dilutions for 10 min at room temperature. The second round of antigen retrieval removed antibody complexes, and tests with the same experimental procedures were performed for the next antibody. After the final round of staining, slides were stained with DAPI for 1 min at room temperature, washed with distilled water, and mounted after air-drying without dehydration. MIHC slides were captured using confocal laser scanning microscopy (LSM800; Carl Zeiss) in a blinded manner and analyzed using ZEN 3.4 (blue edition) software. An experienced pathologist evaluated experimental results.

The primary antibodies used in this study include: AIM2 (1:200, DF3514, Affinity), VIMENTIN (1:200, A19607, Abclonal), EPCAM (1:200, EM1111, HuaBio), CD45 (1:200, A2115, ABclonal), CD11B (1:500, 66519-1-Ig, Proteintech).

### Data acquisition

The transcriptome profiling and clinical information were retrieved from The Cancer Genome Atlas (TCGA) database on the GDC data portal, including 414 bladder tumor samples and 19 normal bladder samples. Ten tumor samples in this queue were not equipped with clinical information. Totally 404 cases of bladder tumor samples and 19 cases of normal samples were finally gathered. Ten tumor samples lacked clinical information. The Toil workflow was applied to normalize the RNA-seq data with TPM format.

### Analysis of immune cell infiltration

The CIBERPORT was utilized to calculate the immune cell infiltration. The principle of the deep learning algorithms was based on convolution and deconvolution. A single sample was treated as a mixture of numerous immune cells to calculate the correlation between the expression of various components in each immune cell and the final combination with linear regression. The expression features of each immune cell were retrieved using the deconvolution technique to obtain the abundance of immune cells linked with the expression matrix.

### Statistical analysis

The statistical analyses are described in the figure legends. The group means for different treatments were compared by ANOVA with Bonferroni’s multiple comparisons test or by a two-tail Student’s *t*-test. For survival analysis, the *p* value was determined by log rank (Mantel-Cox) test. Hazard ratio (HR) and 95% confidence interval (CI) were determined by the Mantel-Haenszel method. Statistical analysis was performed using GraphPad Prism 8.0. Error bars show the mean ± SEM or mean ± SD, as indicated. *p < 0.05, **p < 0.01, ***p < 0.001; ns, not significant (*p* > 0.05).

## Results

### The expression level of innate immune sensor AIM2 positively correlates with the prognosis of the bladder cancer patients

**FIGURE 1:**
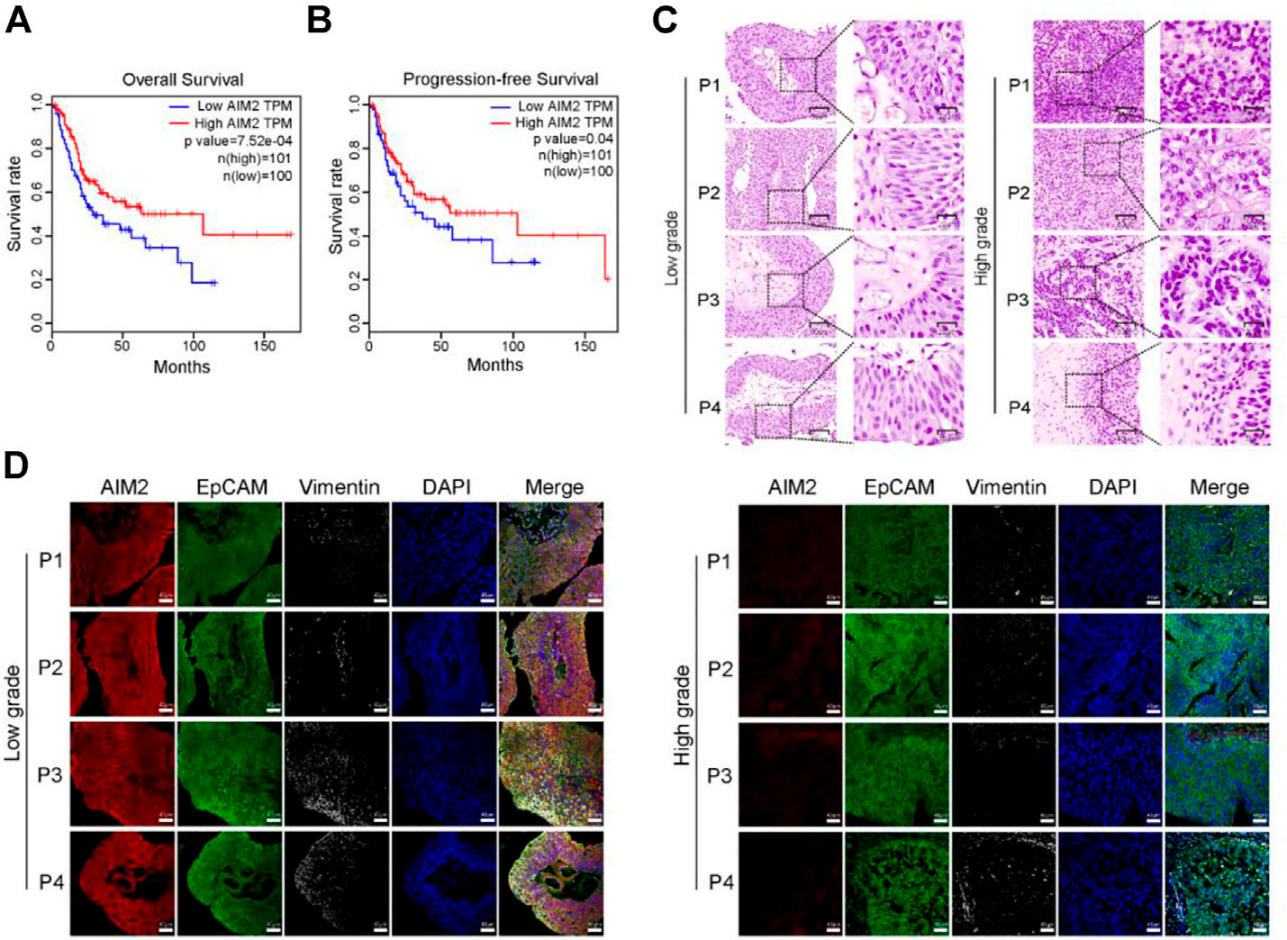
The innate immune sensor AIM2 expression level is positively correlated with the prognosis of BLCA patients. **(A)** Kaplan-Meier curves showing the overall survival (OS) time of patients with BLCA in the TCGA cohort. **(B)** Kaplan-Meier curves presenting the progression-free survival (PFS) of patients with BLCA in the TCGA cohort. **(C)** H and E staining of human high- and low-grade BLCA samples. **(D)** The protein level of AIM2, EpCAM (representing the epithelial component of bladder cancer), and Vimentin (the biomarker of the stromal component) by 4-color multiplex immunofluorescence (MIHC) staining assays. Survival rate **(A,B)** is compared by Log-Rank (Mantel-Cox) test.

The Kaplan-Meier survival analysis is conducted to validate the prognostic relevance of the AIM2 inflammasome in BLCA. The survival curves illustrated that those with lower expression of AIM2 had worse outcome, manifesting as a shorter overall survival (OS) and progression-free survival (PFS) (Figures 1A,B). The tumor tissues are collected from 4 cases of high-grade and 4 cases of low-grade BLCA patients to ascertain the protein level of AIM2 in BLCA (Figure 1C). The protein level of AIM2 in high-grade BLCA was significantly lower than that in low-grade BLCA, suggesting that BLCA may evade inflammasome activation by downregulated AIM2 (Figure 1D). Since NMIBC samples are usually too small and contain a large percentage of stromal components, western blot is not applicable to them. Thus we choose the different BLCA cell lines derived from NMIBC and MIBC to verify differences in AIM2 expression. IB analysis showed that non-invasive cell lines, MGH-U3 and RT4 express higher level of AIM2; while intermediate mesenchymal-like cell line 5637 expresses much lower AIM2 compared to NMIBC lines; moreover, the fully mesenchymal line UMUC3 almost does not express AIM2 (Supplementary Figure S1A). These observations indicate that the innate immune sensor AIM2 is expressed in BLCA cells and helpful effect on the survival of BLCA patients.

### Mice transplanted with AIM2-overexpressed MBT-2 cells indicate prolonged survival and delayed tumor progression

**FIGURE 2:**
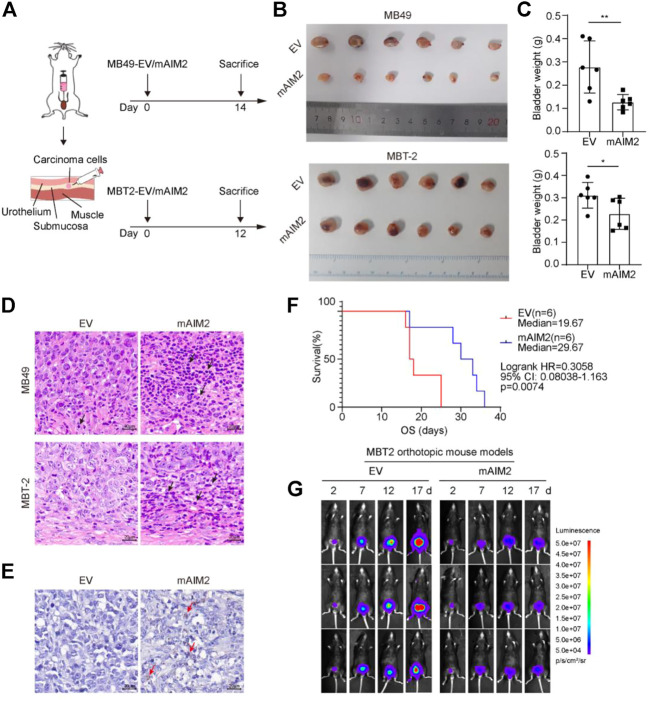
Mice transplanted with mAIM2-overexpressed MBT-2 cells show prolonged survival and delayed tumor progression. **(A)** Experimental schematic. Mice were transplanted with 1.5 × 10^4^ mAIM2-overexpressed MB49 or MBT-2 bladder carcinoma cells and the indicated control cells on day 0 and sacrificed on day 14 or 12, respectively. **(B)** Images of bladders from the indicated groups. **(C)** Bladder weights of orthotopic tumors from the indicated groups. **(D)** Representative pictures of H and E staining of orthotopic MB49 and MBT-2 transplanted BLCA model. Inflammatory cells significantly infiltrate mAIM2-overexpressed tumors. Black arrows indicate inflammatory cells. **(E)** Representative pictures of immunohistochemistry (IHC) staining of CD11b for MBT-2 xenografts. CD11b^+^ cells were significantly enriched in mAIM2-overexpressed tumors. Red arrows indicate inflammatory cells. **(F)** Survival curves of mice transplanted with mAIM2-overexpressed MBT-2 cells or the control. **(G)** Fluorescence of mAIM2-overexpressed MBT-2 cells or the control cells after orthotopic transplantation were monitored by IVIS at indicated days. Data in **(C)** are presented as the means ± SD. *p*-values in **(C)** were calculated using the student’s *t*-test. Survival rate **(F)** is compared by Log-Rank (Mantel-Cox) test. * indicates *p* < 0.05, **, *p* < 0.01, ***, *p* < 0.001, ****, *p* < 0.0001; ns, not significant (*p* > 0.05).

Taking into account that the overexpression of AIM2 may have a beneficial effect on survival, we investigated the effect of AIM2 on BLCA progression using a mouse model. Murine BLCA cells MB49 and MBT-2 stably transduced with AIM2 or empty vector (EV) control (Supplementary Figure S1B) were used for orthotopic transplantation; details are available in (Lorenzatti Hiles et al., 2019). AIM2-overexpressed MB49 cells (2 × 10^4^) or MBT-2 cells (1.5 × 10^4^) and the control cells were injected into the submucosa layer of the bladder of C57BL/6 mice with a surgical orthotopic approach (Figure 2A). At the end of the experiments (14 days for MB49 and 12 days for MBT-2), the AIM2-overexpressed xenografts were much smaller in size and lower in weight than their control counterparts (Figures 2B,C). H&E staining revealed that more inflammatory cells infiltrated the microenvironment of AIM2-overexpressed tumor (Figure 2D). To confirm the effect of AIM2 in regulating immune cell recruitment, the CIBERSORT database (Chen et al., 2018) was used to analyze the association of AIM2 expression and infiltration of immune cell in BLCA patients. Patients with lower protein level of AIM2 showed significantly lower infiltration in several immune cells, including CD4^+^ T-cells, macrophages M1, and dendritic cells (Supplementary Figure S2). Consistently, AIM2 overexpression enhances the recruitment of CD11b^+^ cells (myeloid marker, including neutrophils, monocytes, macrophages, NK cells, and so forth) (Figure 2E). Furthermore, prolonged survival time was observed among mice transplanted with AIM2-overexpressed cells compared to mice with control cells. The median survival time increased by about 1/3 (10 days) (Figure 2F). Fluorescence imaging revealed that the growth rate of AIM2-overexpressed tumors was significantly slower than that of controls (Figure 2G). However, there was no significant difference between AIM2-overexpressed MBT-2 tumors and the control in immune-deficient mice (NCG) (Supplementary Figures S1C–E), suggesting that the survival benefit of mice transplanted with AIM2-overexpressed MBT-2 cells may depend on AIM2-associated immune response.

### AIM2 inflammasome could activate in bladder cancer cells

**FIGURE 3:**
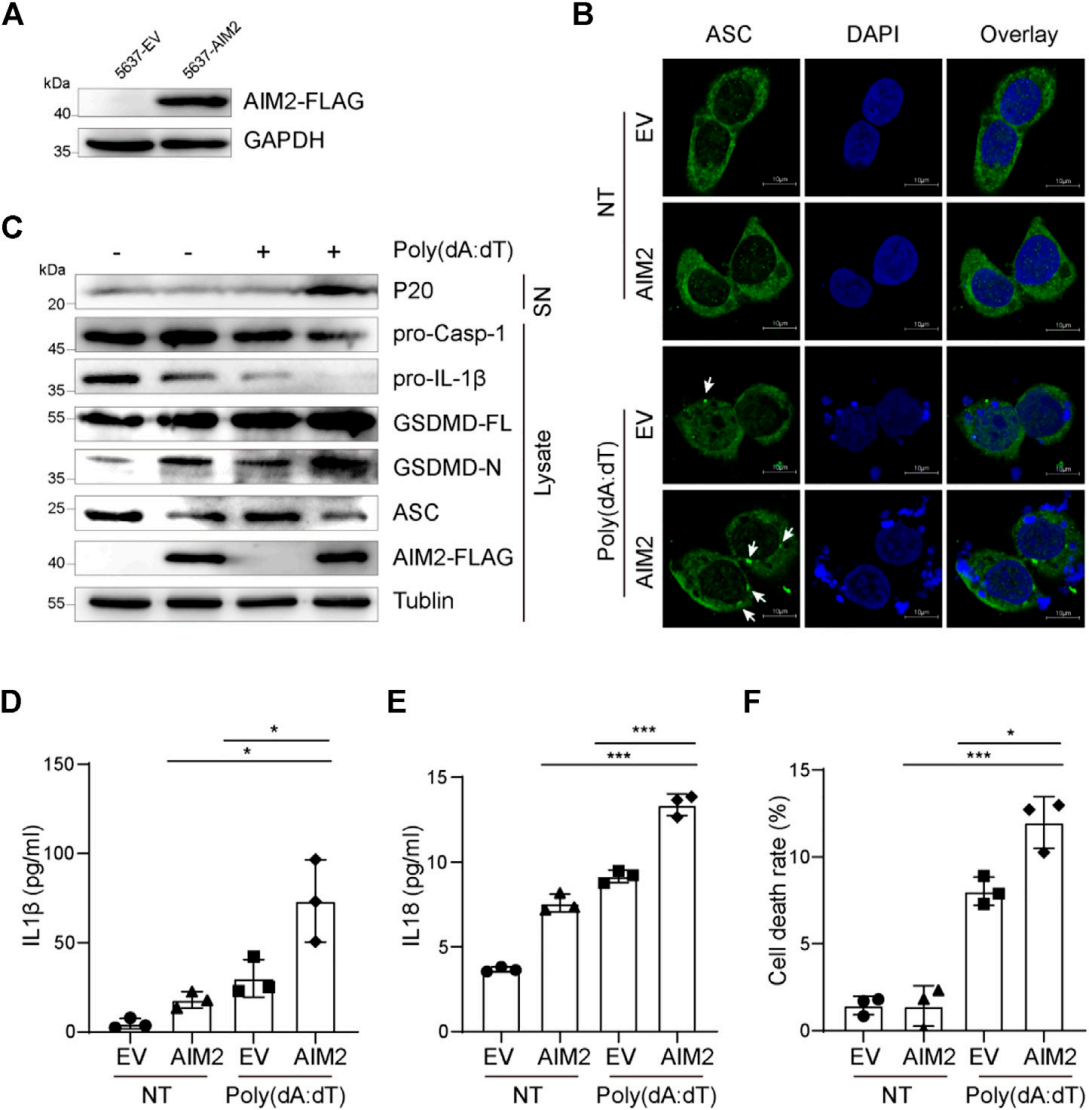
AIM2-overexpression in 5637 cells enhanced inflammasome activation. **(A)** Immunoblotting showing protein level of flag-tagged AIM2 in AIM2-overexpressed 5637 cells and the control. **(B)** Immunofluorescence images of AIM2-overexpressed 5637 cells and the control after transfected with or without poly (dA:dT) for 3 h. White arrows indicate the ASC specks. **(C)** Immunoblot analysis of p20 in the supernatants (SN), and pro-caspase-1, pro-IL-1β, and GSDMD in the lysates of poly (dA:dT)- treated 5637 cells. **(D)** ELISA of IL-1β in the supernatants of 5637 cells after poly (dA:dT) stimulation. **(E)** ELISA of IL-18 in the supernatants of 5637 cells after poly (dA:dT) stimulation. **(F)** Lactate Dehydrogenase (LDH) release in the supernatants of poly (dA:dT) stimulated 5637 cells, detected by diaphorase catalyzed iodonitrotetrazolium chloride (INT) color reaction. In **(D–F)**, the cells were treated with poly (dA:dT) (1 μg/ml, 9 h) or not. Data in **(D–F)** are presented as the means ± SEM. *p*-values in **(D–F)** were calculated using the student’s *t*-test. * indicates *p* < 0.05, **, *p* < 0.01, ***, *p* < 0.001, ****, *p* < 0.0001; ns, not significant (*p* > 0.05).

Since the elevated anti-tumor effect by up-regulated AIM2 depends on AIM2-associated immune response and AIM2 could initiate the immune response by forming an AIM2 inflammasome to release inflammatory cytokines, a hypothesis is proposed regarding AIM2 may act as a tumor suppressor in an AIM2-inflammasome-dependent manner. To verify this hypothesis, we sought to activate the AIM2 inflammasome in BLCA cells. Because the expression of AIM2 was decreased in high-grade BLCA compared to low-grade (Figure 1C), we speculate that AIM2 is detrimental to the progression of BLCA from NMIBC to MIBC. Hence, intermediate mesenchymal-like BLCA cells were selected for experiments *in vitro*. Plasmid expressing AIM2 or EV was transduced to 5637 cells, an intermediate mesenchymal-like BLCA cell line, to generate AIM2 overexpressed 5637 cells and the control cells (Figure 3A). To demonstrate the ability of AIM2 inflammasome activation in BLCA cells, 5637 cells were treated with poly (dA:dT), an AIM2 agonist. Poly (dA:dT) exposure triggered ASC specks in 5637 cells, and the speck increases significantly in the AIM2-overexpressed 5637 cells compared to that of the control (Figure 3B), suggesting an inflammasome activation in 5637 cells (Rathinam et al., 2012). As we known, the AIM2 inflammasome activation could lead to caspase-1 activation, which could further promote the maturation and secretion of IL-1β and IL-18 (Kumari et al., 2020). IB and ELISA analysis illustrated that poly (dA:dT) could induce the processing of caspase-1, pro-IL-1β, and pro-IL-18 in 5637 cells. A decrease of the pro-caspase-1 and pro-IL-1β in the cell lysis and an increase of the cleaved caspase-1 (p20) (Figure 3C) and inflammatory cytokines (IL-1β and IL-18) in the supernatant are detected (Figures 3D–E). In addition to cytokines processing, AIM2 activation leads to a GSDMD-dependent inflammatory form of programmed cell death known as pyroptosis (Rathinam et al., 2010). The AIM2-overexpressed cells present a higher level of GSDMD cleavage and a stronger pyroptosis revealed by LDH release than the control cells (Figures 3C,F). Taken together, our results indicate that AIM2 overexpression augments inflammasome activation triggered by poly (dA:dT) to induce IL-1β and IL-18 secretion and pyroptosis in BLCA cells.

### A151 inhibits the activation of the AIM2 inflammasome in bladder cancer cells

**FIGURE 4:**
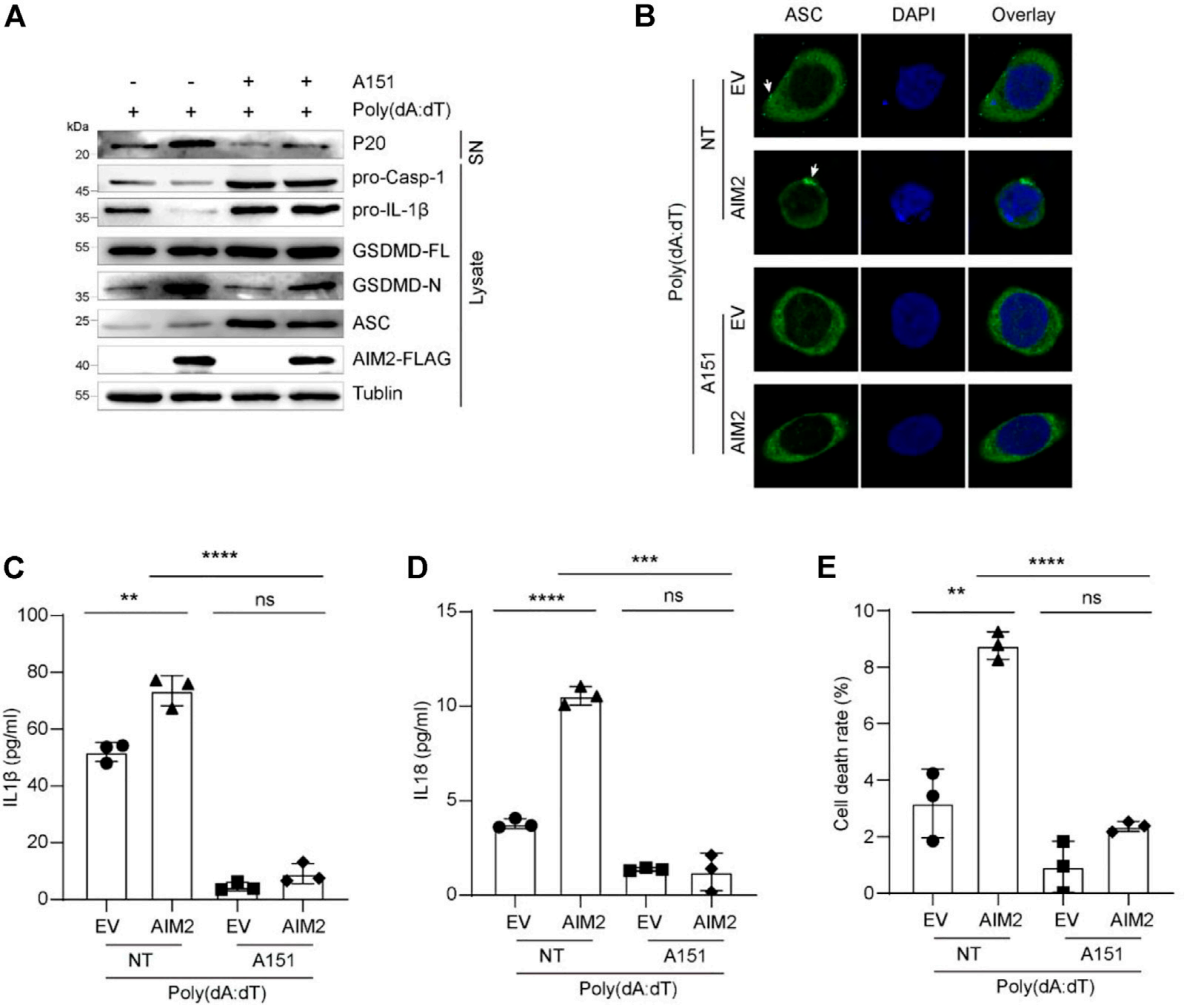
A151 inhibits the activation of AIM2 inflammasome *in vitro*. **(A)** Immunoblot analysis of p20 in the supernatants, and pro-caspase-1, pro-IL-1β, and GSDMD in the lysates of 5637 cells. **(B)** IF images of AIM2-overexpressed 5637 cells and the control with or without A151 inhibition before being transfected with poly (dA:dT). White arrows indicate the ASC specks. **(C)** ELISA of IL-1β in the supernatants of 5637 cells with or without A151 inhibition. **(D)** ELISA of IL-18 in the supernatants of 5637 cells with or without A151 inhibition. **(E)** LDH release in the supernatants of A151 treated 5637 cells and the control. The cells were pre-treated with A151 (1 μM, 1 h) or not followed by poly (dA:dT) (1 μg/ml, 9 h). Data in **(C–E)** are presented as the means ± SEM. *p*-values in **(C–E)** were calculated using the student’s *t*-test. * indicates *p* < 0.05, **, *p* < 0.01, ***, *p* < 0.001, ****, *p* < 0.0001; ns, not significant (*p* > 0.05).

To confirm the activation of AIM2 inflammasome in BLCA, we used ODN TTAGGG (A151), an AIM2 inflammasome inhibitor, which can abrogate activation of cytosolic nucleic acid-sensing pathways and prevent AIM2 inflammasome complex formation (Kaminski et al., 2013). We firstly examined the ability of A151 to modulate AIM2-induced inflammation in 5637 cells. Therefore, 5637 cells were treated with poly (dA:dT) in the presence or absence of A151. IF and IB analysis showed that the decrease of pro-caspase-1, pro-IL-1β, and elevation of ASC specks, activated caspase-1 and mature IL-1β caused by overexpression of AIM2 were abolished by A151 treatment (Figures 4A,B). Compared with the control groups, A151 decreased AIM2 inflammasome activation by approximately 80%, including the release of IL1 and IL18 (Figures 4C,D). Furthermore, treatment with A151 followed by poly (dA:dT) exposure prevented GSDMD activation resulting in cell death (Figures 4A,E), indicating that A151 blocks AIM2-mediated pyroptosis in addition to cytokines maturation. These findings suggest that AIM2-mediated inflammasome signaling works in 5637 cells, demonstrating that the AIM2 inflammasome is functional in BLCA.

### AIM2 overexpression enhances the therapeutic efficacy of BCG immunotherapy

BCG immunotherapy is intravesical immunotherapy based on the bacteria of mycobacterium bovis (bovine TB) and has been used since 1976 to treat BLCA (Morales et al., 1976). Although BCG therapy is the most successful microbial therapy for cancers in current clinical applications (Forbes et al., 2018), it only works in a part of patients with BLCA. How to enhance the efficacy of BCG to reduce recurrence and malignant progression has long bedeviled urologists. The AIM2 inflammasome is known to sense double-stranded DNA (dsDNA), which is abundant within the tumor microenvironment (Sharma et al., 2019; Lammert et al., 2020) and shows a strong positive correlation with immune cell infiltration (Supplementary Figure S2). Therefore, we anticipated that upregulation of AIM2 could improve the effect of BCG immunotherapy.

**FIGURE 5:**
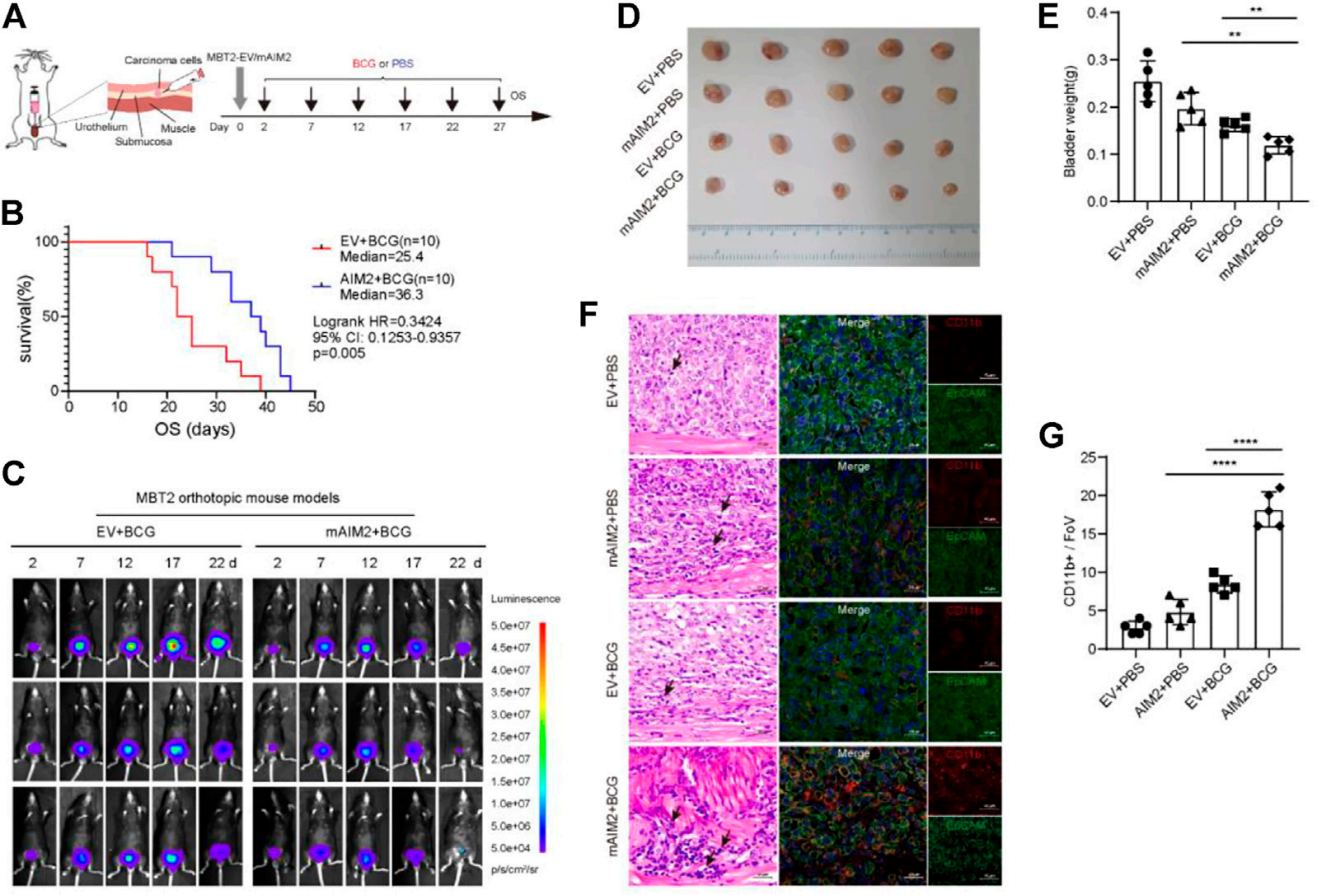
Tumors generated by mAIM2-overexpressed MBT-2 cells were more sensitive to BCG immunotherapy. **(A)** Experimental schematic. Mice were transplanted with 1.5 × 10^4^ mAIM2-overexpressed MBT-2 cells or the control cells on day 0 and receive intravesical BCG or PBS on days 2, 7, 12, 17, 22, and 27. **(B)** Survival curves of mice from the indicated groups. **(C)** *In vivo* bioluminescence images to track the growth of mAIM2-overexpressed MBT-2 cells and the control cells after intravesical BCG instillation of their bladders on days 2, 7, 12, 17, 22, and 27. **(D)** Images of tumors from the indicated groups. **(E)** Bladder weight of orthotopic tumors from the indicated groups. **(F)** Representative images for H and E staining of mice bladders after BCG therapy and confocal micrographs showing CD11b^+^ cells infiltration. Black arrows indicate inflammatory cells. **(G)** Quantification of CD11b^+^ cells infiltration (*n* = 5 Fovs (40×); Fov, field of view). Data in **(E,G)** are presented as the means ± SD. *p*-values in **(E,G)** were calculated using the student’s *t*-test. Survival rate **(B)** is compared by Log-Rank (Mantel-Cox) test. * indicates *p* < 0.05, **, *p* < 0.01, ***, *p* < 0.001, ****, *p* < 0.0001; ns, not significant (*p* > 0.05).

We firstly confirmed that BCG effectively prolonged the survival of mice in orthotopic transplantation model (Supplementary Figures S3A,B), in accordance with previous report (Antonelli et al., 2020). After six rounds of intravesical treatments of BCG every 5 days, a prolonged survival was observed among mice inoculated with AIM2-overexpressed cells compared to control mice: after 30 days post-inoculation, seven out of ten control tumor-bearing mice died, whereas only two out of ten AIM2-overexpressed tumor-bearing mice died (Figures 5A,B). Fluorescence imaging at different times indicated that the tumor sizes were similar at the beginning of orthotopic transplantation. In contrast, after multiple BCG treatments, the fluorescence signals of tumors generated by the AIM2-overexpressed cells significantly decreased compared to the control’s (Figure 5C).

To further characterize the effect of AIM2 overexpression on BCG response, the experiments in MBT-2 cells with or without AIM2 overexpression were repeatedly performed (Supplementary Figure S3C). After 13 days post-inoculation, fluorescence imaging showed that fluorescence intensity in the tumors generated by AIM2-overexpressed cells was less than half of the fluorescence intensity of the control (Supplementary Figure S3D). The mice were sacrificed to harvest the bladders the next day. The tumors generated by AIM2-overexpressed cells and treated by BCG were much smaller than their control counterparts (Figures 5D,E). These results confirmed our finding that AIM2 inhibits BLCA progression and enhances the efficacy of BCG therapy. Moreover, we observed that inflammatory cells were significantly enriched in tumors generated by AIM2-overexpressed cells and treated by BCG by H&E staining (Figure 5F). Furthermore, BCG treatment resulted in a robust infiltration of CD11b^+^ cells, suggesting BCG could activate AIM2 inflammasome and recruited more inflammatory cells to suppress the tumors (Figure 5F). Remarkably, AIM2-overexpression enhanced CD11b^+^ cells recruitment in combination with BCG (Figure 5F). Confocal analysis also revealed that tumors generated by AIM2-overexpressed cells and treated by BCG presented a higher percentage of CD11b^+^ cells (Figure 5G). Collectively, our data indicate that activation of AIM2 inflammasome could improve the curative effect of BCG by increasing CD11b^+^ cells infiltration in the tumor microenvironment to achieve antitumor effects.

## Discussion

Inflammatory responses play complex roles at different stages of tumor development, and efficacy of anticancer therapy that antitumorigenic and protumorigenic inflammatory mechanisms coexist. Inflammasome complex represents the acute inflammatory response of cells to pathogen-associated molecular patterns (PAMPs) and damage-associated molecular patterns (DAMPs). As the expressions and functions of different inflammasomes vary among different types of cancers, it is imperative to investigate the role of the inflammasome in BLCA, in which the potential roles of inflammasomes have not been elucidated. In this study, we demonstrate that AIM2 in BLCA cells, but not bone marrow cells, restricts BLCA progression through the inflammasome-dependent mechanism. Moreover, it is also suggested that AIM2 inflammasome activation enhances the therapeutic effect of BCG in BLCA.

Inflammasomes, potent inducers of IL-1β and IL-18 and pyroptosis, act as double-edged swords in cancers by producing tumor-promoting proinflammatory cytokines or facilitation of antitumor immune responses (Kolb et al., 2014). The AIM2 inflammasome activation by both exogenous and endogenous dsDNA is one of the most critical inflammasomes. In different types of cancer, AIM2 also plays a pro-oncogenic or anti-cancerous role. For instance, AIM2 is highly expressed in NSCLC and AIM2 inflammasome activation promotes the growth of NSCLC cells. In addition, inhibition of AIM2 could contribute to NSCLC therapy (Yu et al., 2019; Zhang et al., 2019). While the high expression of AIM2 is beneficial to the survival of tumor patients in hepatocellular carcinoma (HCC) (Chen et al., 2017). In nasopharyngeal carcinoma (NPC), AIM2-induced IL-1β in tumor cells plays a crucial role in tumor control by recruiting neutrophils (Chen et al., 2012). In our study, high expression of AIM2 correlates with high survival, and the protein level of AIM2 is significantly higher in low-grade BLCA, suggesting AIM2 is a tumor suppressor. Interestingly, analysis of online databases suggests that BLCA express higher AIM2 compared to normal urothelium (data not shown). These findings innovated us to ask whether we could control BLCA progression through enhancing AIM2 activation. As supposed, AIM2 restricts BLCA progression in an inflammasome-dependent manner *in vitro* and *in vivo*. Once the AIM2 inflammasome is activated by cellular stress or nucleic acid fragments released by dead cells, tumor cells release inflammatory cytokines and recruit CD11b^+^ neutrophils to inhibit tumor growth. Although IL-1β is often considered pro-tumourigenic, especially in chronic inflammatory conditions (Shadpour et al., 2019), inflammasome and IL-1β-mediated antitumor activity have also been demonstrated in NPC and hematopoietic cells, consistent with our foundings (Ghiringhelli et al., 2009; Allen et al., 2010; Chen et al., 2012). Our results highlight that activation of the AIM2 inflammasome in BLCA cells, as that in immune cells, could mediate the anti-tumor activities.

Inflammasome activation could lead to GSDMD-mediated pyroptosis. Different tumors and genetic backgrounds respond differently to pyroptosis (Xia et al., 2019). On the one hand, tumor cell pyroptosis inhibits the development of tumors; on the other hand, as a type of proinflammatory death, pyroptosis may lead to excessive inflammation and promote tumor growth (Xia et al., 2019; Fang et al., 2020). Chen et al. (2021) analyzed 2,214 BLCA samples and found phenotypes with high expression of pyroptosis-related molecules are “hot tumors” with better immune function. Moreover, patients with high levels of pyroptosis are more sensitive to chemotherapeutics such as cisplatin and gemcitabine and have a better prognosis. Our findings indicate that AIM2-induced pyroptosis occurs in BLCA cells, and overexpression of AIM2 leads to increased pyroptosis and repressed tumor progression, suggesting that pyroptosis restricts BLCA development, similar to that in HCC, colorectal cancer (CRC) and gastric cancer (GC) (Allen et al., 2010; Williams et al., 2015; Chu et al., 2016; Qiu et al., 2017). Recent research has attempted to combine pyroptosis regulation with various tumor treatments to inhibit tumor development (Fang et al., 2020), suggesting that a pyroptosis switch could be applied in BLCA therapy.

**FIGURE 6:**
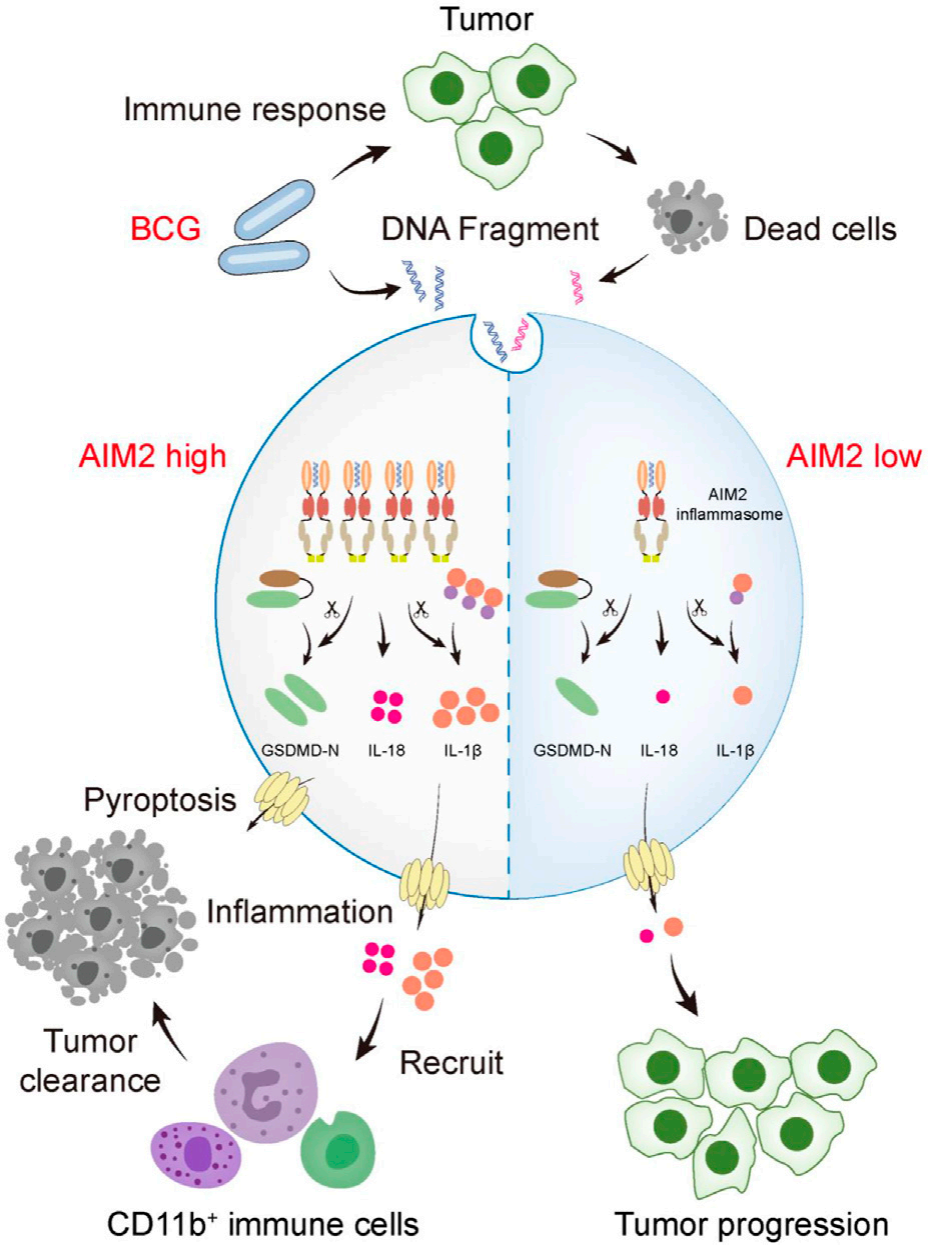
A schematic model that AIM2 inflammasome promotes the sensitivity of BCG therapy in BLCA. During intravesical BCG instillation of mouse bladders, the DNA fragments of BCG and dead tumor cells gain access to the cytosol and are sensed by AIM2. Overexpressed AIM2 assembles more ASC-caspase-1 inflammasome, which activates more IL-1β, IL-18, and GSDMD. The activated GSDMD (GSDMD-N) forms pores on the plasma membrane, causing pyroptosis. Inflammation activation elevates the recruitment of CD11b^+^ cells (myeloid marker, including neutrophils, monocytes, macrophages, NK cells, and so forth) to inhibit the tumor progression. However, cells expressed low level of AIM2 fails to secrete sufficient cytokines to recruit immune cells, leading to tumor progression.

Currently, BCG immunotherapy is the gold-standard treatment for NMIBC with a high risk of progression (Pettenati and Ingersoll, 2018). Although the exact mechanism of BCG immunotherapy is uncertain, it is generally believed that BCG acts as an immunomodulator to destroy tumor cells (Han et al., 2020). BCG could activate the pattern recognition receptor (PRR) to initiate an immune response and then cause cell apoptosis, cell necrosis, and oxidative stress to induce tumor cell death or induce an immune cascade to facilitate the host’s immune system to kill tumor cells (Lobo et al., 2021). Our study found that AIM2 overexpression enhances the therapeutic efficacy of BCG immunotherapy in mice, with a more robust infiltration of CD11b^+^ neutrophils, smaller tumor size and weight, and longer survival time. On the one hand, both AIM2 overexpression and BCG perfusion alone can lead to tumor cell death and anti-tumor immunity. On the other hand, the release of nuclear and mitochondrial DNA by BCG-induced cell death further activates the AIM2 inflammasome, thereby aggravating tumor cell pyroptosis and promoting the production of inflammatory factors to inhibit the tumor growth. Meanwhile, AIM2, as a PRR, could be activated by BCG-induced immune cascade, which elevated the immune response level of BCG. This additive effect results in the better efficacy of BCG immunotherapy in AIM2-high-expressed bladder tumors (Figure 6).

In summary, our findings demonstrate a potent antitumor role of AIM2 inflammasome activation and reveal the feasibility of combined strategies for AIM2 inflammasome activation and intravesical BCG perfusion therapy for BLCA treatment. Moreover, we found that inflammatory cytokines and pyroptosis are crucial tumor suppressors in BLCA. Our study proposes a mechanism that AIM2 suppresses tumor progression and suggests AIM2 inflammasome activation during BCG treatment is a potential strategy for patients with BLCA.

## Data Availability

The original contributions presented in the study are included in the article/Supplementary Material, further inquiries can be directed to the corresponding authors.
