# Supplementary material for: AIM2 inflammasome activation benefits the therapeutic effect of BCG in bladder carcinoma
Source: Front Pharmacol. 2022 Oct 31;13:1050774. doi: 10.3389/fphar.2022.1050774 (PMC9659910; doi:10.3389/fphar.2022.1050774)
Supplement: Supplementary file 1 [file Table1.DOCX]

**Online Supplementary Information**

**AIM2 inflammasome activation benefits the therapeutic effect of BCG in bladder carcinoma**

Houhong Zhou^1,2#^, Lei Zhang^1#^, Weihan Luo^1,2^, Huaishan Hong^3^, Dongdong Tang^1^, Dewang Zhou^1^, Lingli Zhou^1*^, Yuqing Li^1,4*^

^1^Institute of Urology, The Third Affiliated Hospital of Shenzhen University (Luohu Hospital Group), Shenzhen University, Shenzhen 518000, China.

^2^Luohu Clinical Medicine School, Shantou University Medical College, Shantou University, Shantou, 515000, China.

^3^Department of Urology, Fujian Provincial Hospital, Fuzhou, Fujian, 350001, China.

^4^South China Hospital, Health Science Center, Shenzhen University, Shenzhen, 518116, China.

^#^These authors contributed equally.

*** Corresponding Authors:**

Lingli Zhou, Ph.D.

zhoulingli@mail.sysu.edu.cn

Yuqing Li, Ph.D.

liyuqing@email.szu.edu.cn

**Supplementary Figure legends**

Fig. S1 mAIM2-overexpression fails to alter the growth of the xenografts in NCG mice.

Fig. S2 Patients with higher AIM2 expression enhanced cytotoxic immune cell recruitment.

Fig. S3 mAIM2-overexpression re-sensitizes MBT-2 cells to BCG immunotherapy in the orthotopic transplantation BLCA model.

**
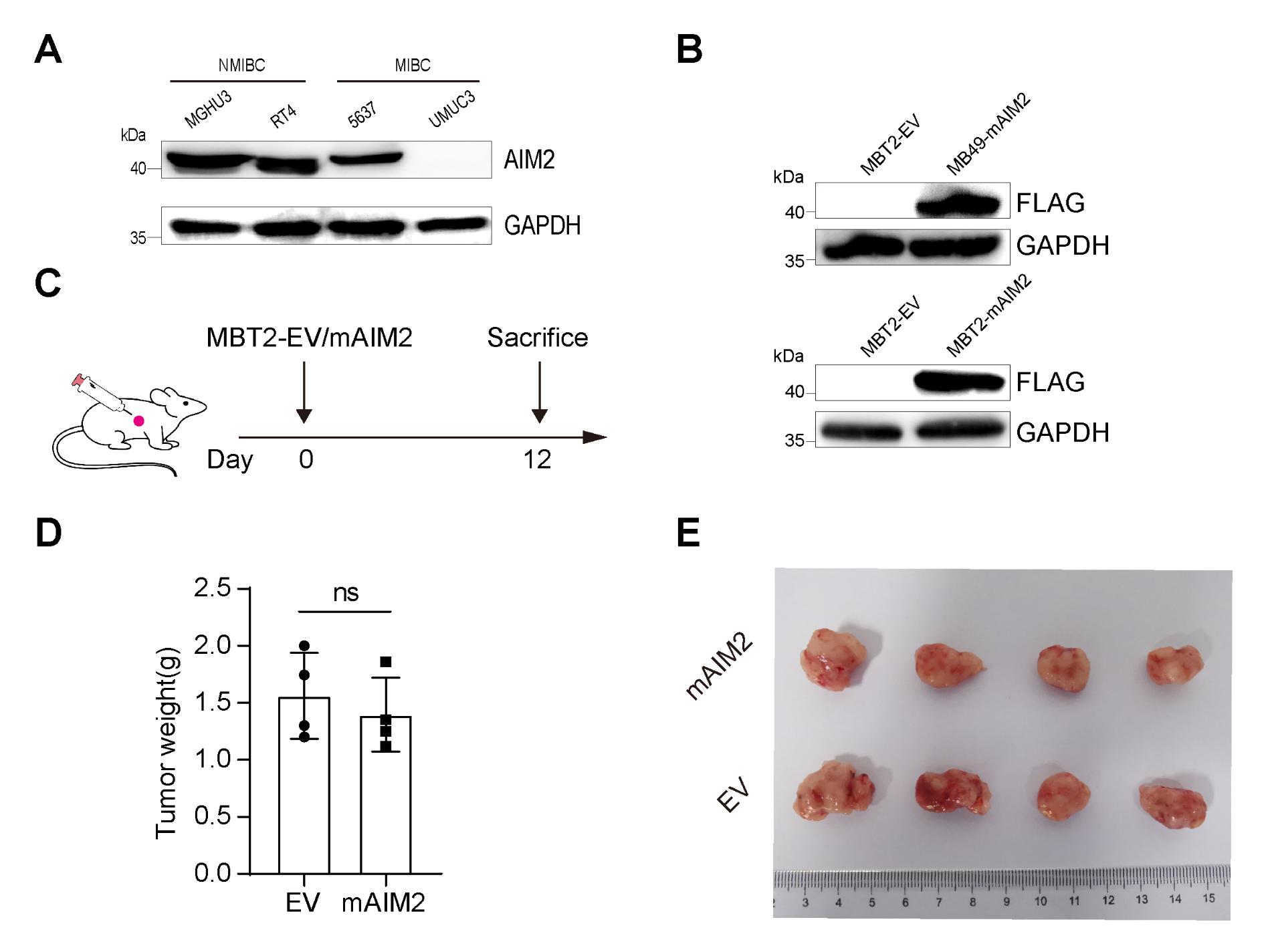
**

**Fig. S1 mAIM2-overexpression fails to alter the growth of the xenografts in NCG mice**.

1. Immunoblotting showing AIM2 expression in different BLCA cell lines.

(B) Immunoblotting showing the protein level of flag-tagged mAIM2 in mAIM2-overexpressed MB49 or MBT2 cells and the control.

(C) Experimental schematic. Mice were subcutaneous transplanted with 1×10^6^ indicated cells on day 0 and sacrificed on day 12.

(D) The weight of tumors measured when mice were sacrificed.

(E) Xenografts in each group.

Data in (D) are presented as the means ± SD. *P*-values in (D) were calculated using the student’s t-test. * indicates *p* < 0.05, **, *p* < 0.01, ***, *p* < 0.001, ****, *p* < 0.0001; ns, not significant (*p* > 0.05).

**
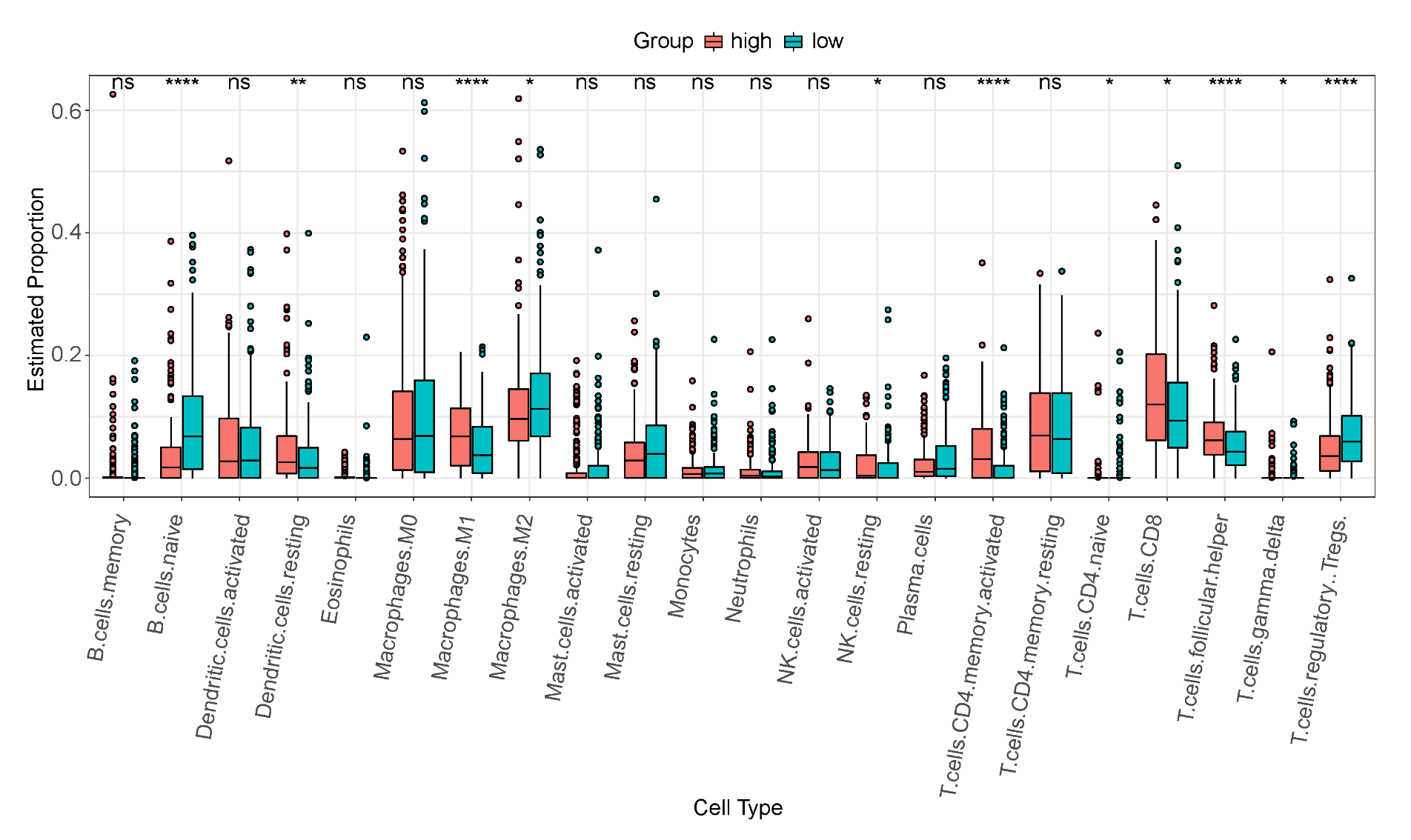
Fig. S2 Patients with higher AIM2 expression show enhanced cytotoxic immune cell recruitment.** CIBERSORT algorithm evaluated the infiltration of the immune cells in high-AIM2 (n=216) and low-AIM2 (n=215) expression BLCA patients in the CIBERSORT database (https://cibersortx.stanford.edu/index.php), which compared by Wilcox. Test, * indicates *p* < 0.05, **, *p* < 0.01, ***, *p* < 0.001, ****, *p* < 0.0001; ns, not significant (*p* > 0.05).

**
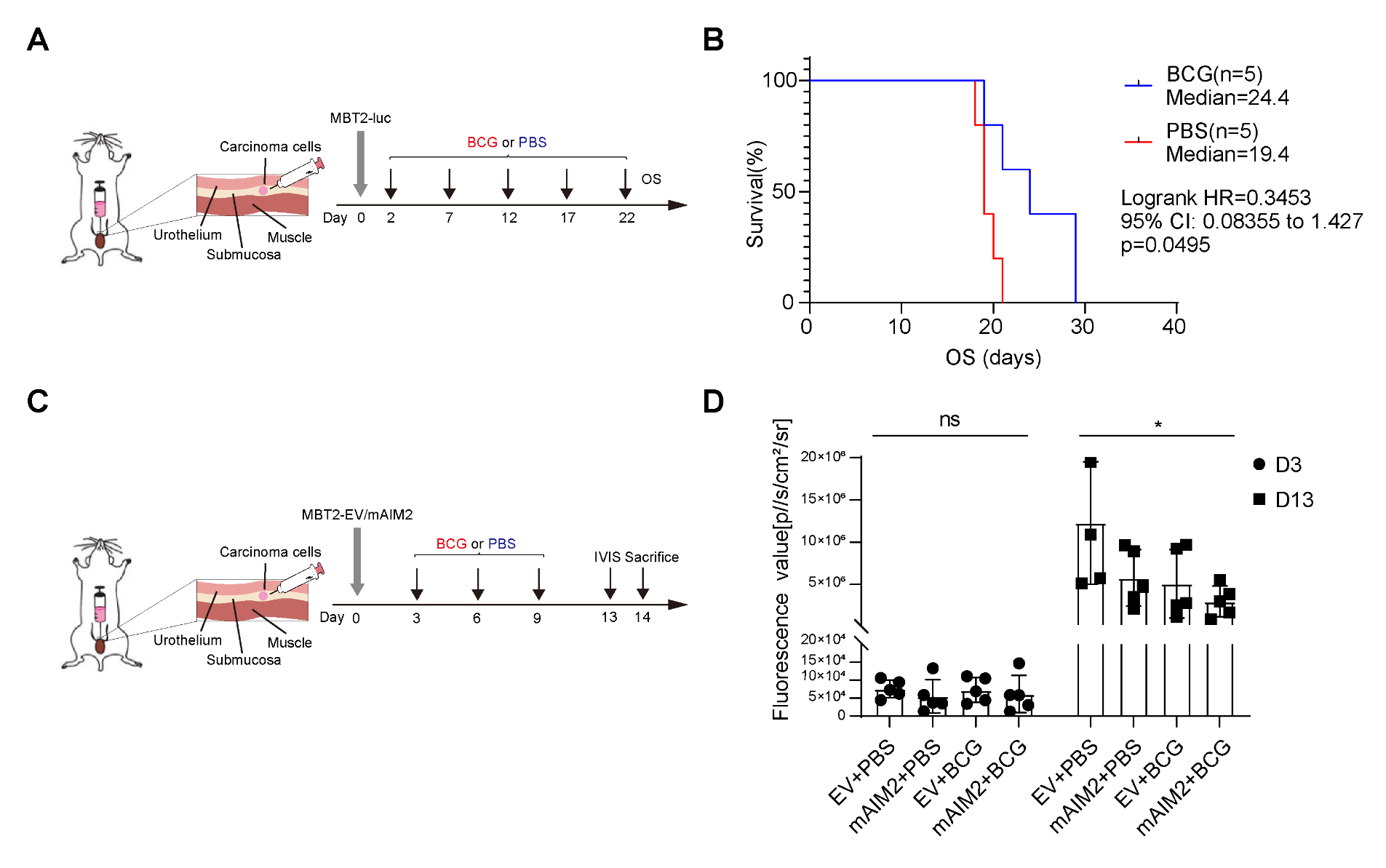
Fig. S3 mAIM2-overexpression promotes the sensitivity of MBT-2 cells to BCG immunotherapy in the orthotopic transplantation BLCA model.**

(A) Experimental schematic. Mice were transplanted with 1.5×10^4^ MBT2-luc cells on day 0 and received intravesical BCG or PBS on days 2, 7，12，17, and 22.

(B) Survival curves of mice transplanted with MBT2-luc cells, treated with BCG or PBS.

(C) Experimental schematic. Mice were transplanted with 1.5×10^4^ control or mAIM2-overexpressed MBT2 cells on day 0 and received intravesical BCG or PBS at the indicated point (Group 1: EV+PBS, Group 2: mAIM2+PBS, Group 3: EV+BCG, Group 4: mAIM2+BCG).

(D) The bioluminescence signal intensities of tumors during intravesical treatment on days 3 and 13 from the indicated groups.

Data in (D) are presented as the means ± SD. *P*-values in (D) ANOVA with Bonferroni’s multiple comparisons test. Survival rate (B) is compared by Log-Rank (Mantel-Cox) test. * indicates *p* < 0.05, **, *p* < 0.01, ***, *p* < 0.001, ****, *p* < 0.0001; ns, not significant (*p* > 0.05).
